# A Pilot Study on Parameter Setting of VisiTag™ Module during Pulmonary Vein Isolation

**DOI:** 10.1155/2018/8960941

**Published:** 2018-10-29

**Authors:** Yu-Chuan Wang, Bo Huang, Kang Li, Peng-Kang He, Er-Dong Chen, Yu-Long Xia, Jie Jiang, Qin-Hui Sheng, Jing Zhou, Yan-Sheng Ding

**Affiliations:** ^1^Department of Geriatrics, Peking University First Hospital, Beijing, China; ^2^Department of Cardiology, Peking University First Hospital, Beijing, China

## Abstract

**Objectives:**

To identify optimal predefined criteria (OPC) for filters of the VisiTag™ module in the CARTO 3 system during pulmonary vein isolation (PVI).

**Methods:**

Thirty patients with atrial fibrillation (AF) who experienced PVI first were enrolled. PVI was accomplished by using a Thermocool SmartTouch catheter. Ablation lesions were tagged automatically as soon as predefined criteria of the VisiTag™ module were met. OPC should be that ablation with the setting resulting in the conduction gap (CG) as few as possible, while contiguous encircling ablation line (CEAL) without the tag gap (TG) on the 3D anatomic model as much as possible.

**Result(s):**

When ablation with parameter setting is being catheter movement with a 3 mm distance limit for at least 20 s and force over time (FOT) being off, there were 60 CEAL without TG on the 3D anatomic model. However, 26 CGs were found. After changing FOT setting to be a minimal force of 5 g with 50% stability time, 22 TGs were displayed. Of them, 20 TGs were accompanied by CGs. On reablation at sites of TG with changed parameter setting, 18 CGs were eliminated when 20 TGs disappeared. When reablation with FOT is being a minimal force of 10 g with 50% stability time, 6 remaining CGs were eliminated. However, there was no CEAL. With a mean of follow-up 10.93 months, 2 patients with persistent AF suffered AF recurrence.

**Conclusion:**

A 3 mm distance limit for at least 20 s and FOT being a minimal force of 5 g with 50% stability time might be OPC for the VisiTag™ module.

## 1. Introduction

Since pulmonary vein isolation (PVI) has been verified to be the cornerstone for treatment of atrial fibrillation (AF) with radiofrequency ablation, making a durable PVI has always been the goal we are chasing. Despite advances in technology, resumption of conduction between the PV and the left atrium is not uncommon, which plays a critical role for AF recurrence [1, 2]. This should be partly attributed to the lack of an effective method to quantify the efficacy of each ablation application during procedure of PVI. As we all know, without the help of the intelligent assistant system many factors affecting the efficacy of ablation cannot be objectively judged by operators during procedures, such as contact force (CF) and catheter stability. With the advent of the CF sensing catheter and automated ablation lesion tagging software, annotation of ablation lesion can be tagged automatically as soon as the predefined criteria for tagging are met. For this reason, a better result of ablation might be achieved if the tagging system is employed with reasonable predefined criteria.

VisiTag™ module (Biosense Webster, Inc.) is an automated ablation lesion tagging software on the CARTO 3 system (Biosense Webster, Inc.), which can continuously store, track, and quantify ablation catheter positions along with the electrophysiological parameters acquired during RF applications. It contains settable constraints for the catheter status according to user preference, including maximal range of catheter drift, minimal time of catheter-tissue connection, and force over time (FOT) which means percentage of minimal time with CF consistently above certain number of grams, impedance drop, and target temperature. Once all preset values for constraints are met, an annotation of ablation lesion would be automatically put on the 3D electroanatomic model during each application of ablation. Thus, quantifying the efficacy of each ablation application might be reflected indirectly and objectively. To our knowledge, however, there is no any recommendation for predefined criteria of VISITAG™ module for initial ablation of PVI. In this study, we hypothesize that optimal predefined criteria (OPC) should meet the ablation with the setting and should result in both conduction gap (CG) and tag gap (TG) on contiguous encircling ablation line (CEAL) as few as possible.

## 2. Methods

### 2.1. Patient Population

From January 2017 to September 2017, thirty patients with drug refractory paroxysmal or persistent AF underwent catheter ablation with the 3D navigation CARTO 3 system were recruited. Antiarrhythmic drugs except amiodarone were discontinued at least 5 half-lives before the procedure. All these patients provided a written informed consent before procedures.

### 2.2. VisiTag™ Module Setting

VisiTag™ module was employed in all ablation procedures for automated tagging of ablation lesion. The filter of respiration adjustment was ticked for avoiding the effects of breathing on tags. The filters, impedance drop and target temperature, were not ticked because these two filters were not well predicted according to the suggestion by Johnson & Johnson. For local drift of the ablation catheter during ablation, 3 mm was used to define the maximal moving range of the catheter according to the outcome of study by Ullah et al. [3]. Twenty-second was used to define the minimal stability time of the ablation catheter based on the study by Chikata et al. [4]. These two parameters, 3 mm and 20 s, were constant in the study. As for FOT, 3 different settings were used during the procedure. At the beginning, the values of the filters in FOT, time, and minimum force, were zero until accomplishment of isolation line around ipsilateral PVs. After that, 50% for time in conjunction with 5 g for minimum force or 50% for time in conjunction with 10 g for minimum force was used as a FOT setting successively. Diameter of lesion tag size was 4 mm.

### 2.3. Ablation Procedure

All patients underwent the procedure under conscious sedation. Transesophageal echocardiography or computed tomography was performed to rule out thrombus in the left atrium before the procedure. After dual transseptal catheterizations, 3D electroanatomical maps of the LA and PVs were reconstructed with a 20-pole mapping catheter LASSO (Biosense Webster, Inc.) or PentaRay (Biosense Webster, Inc.). PVI was performed in a wide area circumferential ablation pattern using a Thermocool SmartTouch irrigated-tip contact-force sensing radiofrequency ablation catheter (Biosense Webster, Inc.). The lesions were created by sequential point-by-point application of radiofrequency. The default power setting was 15–25 W for the posterior wall and 30–40 W for other regions of the left atrium. For each ablation application, duration of ablation was 40–60 s with a stable contact of catheter-tissue. The ablation lesions were tagged automatically based on predefined criteria on the VisiTag module. After accomplishment of isolation line around ipsilateral PVs, reablation would be applied at the sites with conduction gap (CG) which indicated electric connection between PV and left atrium.

### 2.4. Pulmonary Vein Isolation

Firstly, a wide-area circumferential ablation around ipsilateral PVs was accomplished with automated tagging criteria being catheter movement with a 3 mm distance limit for at least 20 s without FOT setting. After that, electric conversion would be implemented if it was not sinus rhythm. Then, CGs were identified by the mapping catheter. The value of the FOT filters was set to hide the tags which did not conform to FOT criteria. Thus, tag gaps (TGs) would emerge on the 3D electroanatomic map. TG was defined as the distance between two neighboring tag points more than 7 mm from the center point to the center point. Afterwards, PVI was achieved as following strategy: if CG and TG were located at the same site of ablation line when FOT setting is being a minimal force of 5 g or 10 g with 50% stability time, elimination of CG was attempted by putting new tags on TG when reablation with FOT setting. If CG still existed after reablation with FOT setting being a minimal force of 10 g with 50% stability time, ablating the corresponding part of pulmonary vein ostium was performed. If TG existed without CG, no reablation was delivered. Bidirection block would be verified after ablation.

### 2.5. Data Analysis

For the purpose of analysis, the ablation line around each ipsilateral PV was divided into 8 distinct segments as previous study [5]. The following data were analyzed after accomplishment of PVI, including force time integral (FTI) and impedance drop (ID) in each ablation application, the distribution of CGs after initial ablation around ipsilateral PVs, and the relationship between CG and TG.

### 2.6. Follow-Up

A 24-hour Holter recording was obtained at the 3 month and 6 month after the procedure. A 12-lead electrocardiogram was assessed at every follow-up. Chronic clinical success at the 6-month follow-up was defined as the absence of sustained AF, atrial flutter, or atrial tachycardia for over 30 s after the blanking period no matter with or without the antiarrhythmic drug.

### 2.7. Statistical Analysis

Continuous variables are expressed as mean and standard deviation. Categorical variables are presented as frequency or percentage. All statistical analysis was performed using SPSS (version 22, IBM Corp., Armonk, NY).

## 3. Results

Of these 30 patients, 24 were male with mean age 58.40 ± 12.11 years. Paroxysmal AF was diagnosed in 23 patients. The anteroposterior diameter of the left atrium on transthoracic echocardiography was 39.54 ± 5.06 mm. The procedure lasted an average time of 161.34 ± 34.29 minutes, with an average ablation time of 57.78 ± 12.65 min, and a mean fluoroscopy time of 11.48 ± 9.02 min. There were no severe cardiac complications, such as pericardial tamponade and atrioesophageal fistula. With a mean follow-up of 10.93 ± 2.10 months, 2 patients with persistent AF suffered AF recurrence after the 3-month blanking period.

### 3.1. Contact Force, Force Time Integral, and Impedance Drop

A total of 2496 ablation lesions were delivered during the PVI procedures (83.20 ± 10.76 lesions per patient). Mean values of CF, FTI, and ID in initial ablation were shown in Table 1. At this moment, automated tag criteria in the VisiTag™ module was catheter movement with a 3 mm distance limit for at least 20 s without the FOT setting. After reablation for elimination of CG while adding FOT setting minimal force 5 g with 50% stability time to previous criteria for automated tagging, recalculated mean values of CF, FTI, and ID were shown in Table 2.

### 3.2. The Relationship between CGs and TGs Based on Different Tagging Criteria

**Figure 1:**
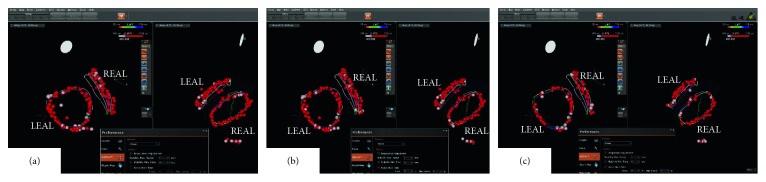
An example of different parameter settings of the VisiTag™ module for pulmonary vein isolation: a 3 mm maximal moving range of the catheter and 20 s minimal stability time of the ablation catheter were constant. (a) FOT setting being zero; (b) FOT setting being minimal force 5 g with 50% stability time; (c) FOT setting being minimal force 10 g with 50% stability time. Different color lines indicated different segments of ablation line. White arrow indicated a TG at the situation of FOT setting being a minimal force of 5 g with 50% stability time, which did not display when FOT setting is zero. FOT: force over time; LEAL: left encircling ablation line; REAL: right encircling ablation line.

Contiguous encircling ablation line (CEAL) consisted of tag points one by one was achieved in all the patients when automated tag criteria being catheter movement with a 3 mm distance limit for at least 20 s without FOT setting. In such situation, there was no TG on CEAL. However, CGs were verified in 26 segments out of 17 pairs of ipsilateral PVs in 11 patients. The distribution of CGs was 2 in the left superior, 6 in the left anterior superior, 7 in the left anterior, 2 in the left anterior inferior, 1 in the left inferior, 3 in the right superior, 1 in the right posterior superior, 2 in the right posterior inferior, 1 in the right inferior, and 1 in the right anterior inferior, respectively. Once FOT setting minimal force 5 g with 50% stability time was included into tagging criteria, 22 TGs displayed on encircling ablation line. Among them, 20 TGs were localized at the sites of CG. They were 1 in the left superior, 4 in the left anterior superior, 5 in the left anterior, 1 in the left anterior inferior, 1 in the left inferior, 3 in the right superior, 1 in the right posterior superior, 2 in the right posterior inferior, 1 in the right inferior, and 1 in the right anterior inferior, respectively. The other two TGs were localized at 1 in the left posterior inferior and 1 in the right anterior inferior, respectively. After reablation with adjusted tagging criteria, 18 CGs were eliminated while 20 TGs were filled by new tag points. When FOT setting was changed to minimal force 10 g with 50% stability time, almost half of the tag points disappeared on the 3D electroanatomic map, and there was no CEAL in all patients (Figure 1). Reablating at such tagging criteria, 6 out of 8 CGs were eliminated with annotation of new tag points. They were localized at 1 in the left superior, 3 in the left anterior superior, and 2 in the left anterior, respectively. The remaining 2 CGs were eliminated when ablation was delivered at the areas of the left anterior carina.

## 4. Discussion

PVI has been verified playing very important role in treatment of AF with radiofrequency ablation [6, 7]. However, duration of PVI was unsatisfactory since recovery of PV conduction had not been an uncommon phenomenon. Multiple reasons might contribute to recovery of PV conduction, including unstable tissue-catheter contact during the ablation and traditional method of annotation of ablation lesion. Without assistance of the intelligent tool, it is hard for the operator to objectively judge the stability of the catheter during ablation application. In addition, traditional ablation lesion tagging bears a strong tinge of subjectivity. Ablation lesion would be annotated as soon as ID or morphological change of the electrogram met the operator's target. After that, the ablation catheter would be rolled to other areas. As a result, inadequate damage for each ablation might be produced. With the employment of the VisiTag™ module, an automated tagging of ablation lesion is realized, which can uniform the standard of ablation lesion annotation and reduce the influence of subjectivity. As a result, a better outcome of PVI could be expected when the VisiTag™ module is employed during the procedure of PVI. In study by Tanaka et al., successful PVI at completion of the initial anatomical line was more frequent when the VisiTag™ module was used for ablation annotation [8]. Similarly, a lower recurrent rate at follow-up was proved when comparison in patients underwent AF ablation with or without employment of the VisiTag™ module [9]. However, a 66.3% successful rate of PVI at the initial procedure and a percentage of 77.5% freedom from atrial tachyarrhythmias at a 12-month follow-up in aforementioned studies might indicate suboptimal parameter settings for filters of the VisiTag™ module, respectively. In study by Fujiwara et al., although the ablation lesions with the acute conduction block was realized at a mean ablation time 12.5 s, the maximum value of FTI in these lesions was 213 g·s, which was almost half of an accepted reference value 400 g·s [10]. For these reasons, what is OPC for filters of the VisiTag™ module is still to be tested. A lenience setting might prevent the endurance of PVI, whereas a strict setting might increase the complication of ablation. To test OPC for the automated tagging system VisiTag™ module during the initial procedure of PVI, we did this study.

According to the results of previous studies, 3 mm maximal moving range of the catheter and 20 s minimal stability time of the ablation catheter were constant in this study. As for FOT, 3 different settings were tested. When ablation with FOT setting is being zero, a CEAL consisting of tag points one by one around ipsilateral PVs was shown on the 3D electroanatomic map in all patients. However, CGs were verified in 26 segments (5%) out of 480 segments in 30 patients. Once FOT setting minimal force 5 g is added with 50% stability time to tagging criteria, 22 segments with TG were displayed. Of these segments, 20 segments localized at the same sites where CGs existed. After reablation with adjusted tagging criteria, 18 CGs were eliminated as soon as TGs were filled by new tag points appeared on the 3D electroanatomic map. So far, PVI was achieved in 26 patients (86.7%) with CEAL on the 3D electroanatomic map. For remaining 8 CGs, 6 of them were eliminated while new tag points were put in TGs during reablation with FOT setting being a minimal force of 10 g with 50% stability time. However, there was no CEAL on the 3D electroanatomic map in all patients at this automated tagging setting. Almost one-third of tag points disappeared from the 3D electroanatomic map. According to this result, we can infer that it is difficult to achieve a CEAL when ablation with tagging criteria being 3 mm maximal moving range of the catheter and 20 s minimal stability time of the ablation catheter with FOT setting being minimal force 10 g with 50% stability time. To many inexperienced operators, however, achievement of CEAL around ipsilateral PVs is the first step for achieving PVI. For this reason, repeated ablations might be implemented by part of operators because it has been proven that human behavior are influenced by the visual effect [11]. If so, unnecessary overablation would be performed, which could increase the possibility of complication. Thus, with consideration of both efficacy of ablation and effect of automated tagging, the following values might be OPC for the automated tagging system VisiTag™ module during the initial procedure of PVI: 3 mm for maximal moving range of the catheter, 20 s for minimal stability time of the ablation catheter, 5 g for minimal force of FOT, and 50% for stability time of FOT, respectively.

### 4.1. Limitations

This study had some limitations. First, the results are based on a relatively small size of 30 patients. Second, this is the report of a single operator. Third, the ablation procedures were performed under sedation and not general anesthesia with jet ventilation. Forth, an accumulated efficacy for eliminating CG cannot be evaluated due to technical limitations when reablating at sites with CG. Finally, the period of follow-up was not long enough.

## 5. Conclusion

A 3 mm maximal moving range of the catheter and 20 s minimal stability time of the ablation catheter in addition to FOT setting being minimal force 5 g with 50% stability time might be OPC for the automated tagging system VisiTag™ module in the initial ablation procedure of PVI.

## Figures and Tables

**Table 1:** Mean values of CF, FTI, and ID in each ablation segment after the initial wide-area circumferential ablation around ipsilateral PVs without FOT setting.

Segments	CF (g)	FTI (g·s)	ID (Ω)
Left superior	11.88 ± 5.90	512.49 ± 172.83	13.16 ± 6.16
Left anterior superior	8.91 ± 3.73	418.03 ± 148.63	12.15 ± 5.67
Left anterior	10.39 ± 4.18	427.16 ± 163.99	14.51 ± 6.39
Left anterior inferior	9.06 ± 3.49	427.26 ± 153.31	12.55 ± 6.19
Left inferior	11.38 ± 4.44	441.65 ± 152.46	14.02 ± 5.33
Left posterior inferior	14.04 ± 5.68	495.35 ± 174.51	13.13 ± 4.84
Left posterior	11.76 ± 5.15	445.90 ± 143.90	11.24 ± 4.89
Left posterior superior	11.83 ± 5.33	477.17 ± 152.47	12.13 ± 5.58
Right superior	14.29 ± 4.68	552.94 ± 140.85	15.46 ± 6.21
Right anterior superior	14.02 ± 4.63	575.31 ± 149.48	17.67 ± 5.71
Right anterior	17.89 ± 5.65	615.66 ± 153.57	15.85 ± 450
Right anterior inferior	11.56 ± 4.96	514.95 ± 213.81	14.22 ± 8.50
Right inferior	11.81 ± 5.51	482.34 ± 181.52	14.91 ± 6.85
Right posterior inferior	11.92 ± 5.83	511.77 ± 203.11	11.50 ± 4.78
Right posterior	12.96 ± 5.09	517.56 ± 153.14	10.78 ± 4.48
Right posterior superior	13.77 ± 6.92	545.55 ± 239.42	12.00 ± 5.81

CF: contact force; FTI: force time integral; ID: impedance drop.

**Table 2:** Mean values of CF, FTI, and ID in each ablation segment after the initial wide-area circumferential ablation around ipsilateral PVs with FOT setting being minimal force 5 g with 50% stability time.

Segments	CF (g)	FTI (g·s)	ID (Ω)
Left superior	12.28 ± 5.58	537.32 ± 148.71	13.58 ± 5.61
Left anterior superior	10.06 ± 3.08	489.00 ± 141.33	12.89 ± 5.16
Left anterior	11.19 ± 3.57	491.10 ± 137.84	15.41 ± 5.51
Left anterior inferior	9.68 ± 3.01	467.32 ± 144.73	13.00 ± 5.66
Left inferior	11.65 ± 4.17	456.23 ± 137.10	14.21 ± 5.04
Left posterior inferior	14.04 ± 5.68	495.35 ± 174.51	13.13 ± 4.84
Left posterior	11.76 ± 5.15	445.90 ± 143.90	11.24 ± 4.89
Left posterior superior	11.83 ± 5.33	477.17 ± 152.47	12.13 ± 5.58
Right superior	14.53 ± 4.22	557.19 ± 130.73	15.61 ± 5.96
Right anterior superior	14.02 ± 4.63	575.31 ± 149.48	17.67 ± 5.71
Right anterior	17.89 ± 5.65	615.66 ± 153.57	15.85 ± 450
Right anterior inferior	11.90 ± 4.65	536.34 ± 197.24	14.46 ± 8.27
Right inferior	12.36 ± 5.03	514.32 ± 157.16	15.36 ± 6.26
Right posterior inferior	12.96 ± 4.79	559.48 ± 167.37	12.19 ± 4.26
Right posterior	12.96 ± 5.09	517.56 ± 153.14	10.78 ± 4.48
Right posterior superior	14.09 ± 6.63	565.96 ± 229.08	12.27 ± 5.53

CF: contact force; FTI: force time integral; ID: impedance drop.

## Data Availability

The data used to support the findings of this study are available from the corresponding author upon request.
